# Altering the Binding Properties of PRDM9 Partially Restores Fertility across the Species Boundary

**DOI:** 10.1093/molbev/msab269

**Published:** 2021-09-07

**Authors:** Benjamin Davies, Anjali Gupta Hinch, Alberto Cebrian-Serrano, Samy Alghadban, Philipp W Becker, Daniel Biggs, Polinka Hernandez-Pliego, Chris Preece, Daniela Moralli, Gang Zhang, Simon Myers, Peter Donnelly

**Affiliations:** 1 Wellcome Centre for Human Genetics, University of Oxford, Oxford, United Kingdom; 2 Department of Statistics, University of Oxford, Oxford, United Kingdom

**Keywords:** hybrid sterility, recombination, meiosis, speciation

## Abstract

Sterility or subfertility of male hybrid offspring is commonly observed. This phenomenon contributes to reproductive barriers between the parental populations, an early step in the process of speciation. One frequent cause of such infertility is a failure of proper chromosome pairing during male meiosis. In subspecies of the house mouse, the likelihood of successful chromosome synapsis is improved by the binding of the histone methyltransferase PRDM9 to both chromosome homologs at matching positions. Using genetic manipulation, we altered PRDM9 binding to occur more often at matched sites, and find that chromosome pairing defects can be rescued, not only in an intersubspecific cross, but also between distinct species. Using different engineered variants, we demonstrate a quantitative link between the degree of matched homolog binding, chromosome synapsis, and rescue of fertility in hybrids between *Mus musculus and Mus spretus*. The resulting partial restoration of fertility reveals additional mechanisms at play that act to lock-in the reproductive isolation between these two species.

Hybrid sterility is a commonly observed phenomenon in nature. It prevents exchange of genetic information between distinct populations, thereby ensuring their reproductive isolation (Abbott et al. 2013). Hybrid sterility is mainly observed in the heterogametic sex (in mammals, males), according to Haldane’s rule (Haldane 1922), and frequently results from meiotic defects, often involving chromosome asynapsis (Bhattacharyya et al. 2013). To better understand this critical evolutionary process, instances of hybrid sterility have been extensively studied (Maheshwari and Barbash 2011; Chen et al. 2016). In mouse, work on intersubspecific crosses between *Mus musculus domesticus and M. musculus musculus* (Forejt and Iványi 1974) has identified *Prdm9* as the first and so far only speciation gene found in mammals (Mihola et al. 2009).

*Prdm9* has two known roles in mammalian meiosis. The first identified role is that PRDM9 controls the positioning of double-strand breaks (DSBs), which initiate recombination between homologous chromosomes during meiosis (Baudat et al. 2010; Myers et al. 2010; Parvanov et al. 2010). This positioning is defined by the DNA-binding zinc finger array of PRDM9, which is highly variable between different individuals, populations, subspecies, and species, with different DNA sequences being recognized (Oliver et al. 2009; Buard et al. 2014). Recent work has established a second role for PRDM9, namely that although recombination is initiated by a DSB on one of the homologs, the binding of PRDM9 at the corresponding position on the other, *uncut*, homolog facilitates homology search, ensuring successful synapsis (Hinch et al. 2019; Li et al. 2019).

Although the mechanisms of this second role remain unknown, both functions underlie hybrid infertility between subspecies. The DNA sequences at recombination sites change over evolutionary time, partly due to chance mutation and partly as a consequence of DSB repair mechanisms (Pratto et al. 2014). These changes are strongly biased in favor of mutations disrupting—eroding—the strongest binding sites for a particular *Prdm9* allele, eliminating many PRDM9 binding sites from the genome (Myers et al. 2010; Baker et al. 2015). Recombination events move to weaker PRDM9 binding sites, maintaining successful chromosome synapsis and meiosis. Due to the first role of PRDM9, in two isolated populations A and B with two distinct *Prdm9* alleles binding different sequences (say, *Prdm9*^A^ and *Prdm9*^B^, respectively), distinct sites erode within each population. The best PRDM9^A^ binding sites are lost from the genomes of individuals in population A but remain intact in the individuals in population B (fig. 1*a*), and conversely. In a hybrid individual, with one genome from each of the populations, and one copy of each *Prdm9* allele, PRDM9^A^ will preferentially bind to the uneroded, strong binding sites, which are retained mainly on the population “B” chromosomes they inherit. Because PRDM9 binding positions recombination-initiating DSBs, events positioned by PRDM9^A^ tend to occur on the “B” chromosome. Importantly, the homologous sites on the “A” chromosome are mainly not bound by PRDM9, due to erosion of these sites in population A. The same will occur, *mutatis mutandis*, for the PRDM9^B^ allele. This compromises successful chromosome synapsis, because of the second role of PRDM9 (fig. 1*b*).

**Fig. 1:**
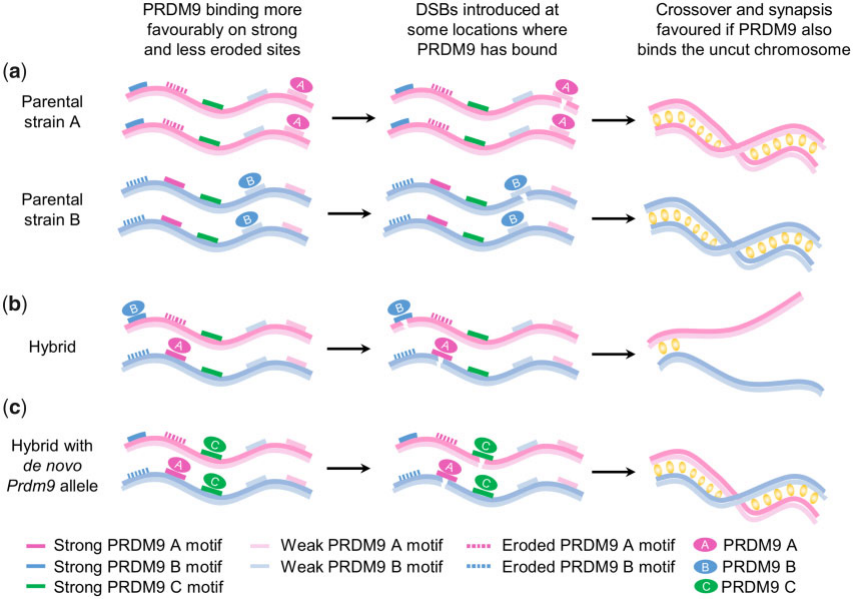
A model of the impact of PRDM9 binding on chromosome synapsis. (*a*) In two parental strains, A and B, different *Prdm9* alleles are present, encoding PRDM9^A^ and PRDM9^B^, which have distinct binding sites, with a spectrum of affinities (ranging from strong to weak). Due to the effects of mutation and recombination, these sites, preferentially the stronger sites, are frequently eroded. PRDM9 will still bind to its intact weaker binding sites and position DSBs to initiate the recombination process. PRDM9 is likely to bind at a similar level at these weaker sites at matched positions on the uncut homolog, enabling synapsis to occur and meiosis to proceed. (*b*) In the hybrid strain, two parental chromosomes and both PRDM9^A^ and PRDM9^B^ are present. The two PRDM9 variants now bind preferentially to the noneroded stronger binding sites on the complementary chromosome, leading to a reduction in cut sites at which PRDM9 is bound to the matched position on the homolog. Chromosome synapsis is inefficient and meiosis arrests. (*c*) In the hybrid, the replacement of one of the *Prdm9* alleles with a de novo allele, *Prdm9^C^* encoding PRDM9^C^ whose distinct binding sites are not eroded on either parental chromosome, leads to strong binding to this noneroded motif on both parental chromosomes, increasing the frequency of PRDM9 binding matched positions, favoring chromosome synapsis, and rescuing the meiotic arrest.

Although the reason(s) this leads to asynapsis are not known, it seems likely that, following DSB formation, the homologous sequence is essential for recombinational repair of the DSB, and differences between homologs within PRDM9 binding motifs thus have the potential to disrupt this process. Indeed, it has been observed that at individual hotspots where one copy of the PRDM9 binding motif is eroded, DSBs occurring at the other, noneroded copy are repaired more slowly, and are less likely to be recombinationally repaired using the homolog (Hinch et al. 2019; Li et al. 2019). This implyies a disruption of homology search and/or homologous repair operating at individual hotspots. Recent work (Huang et al. 2020; Mahgoub et al. 2020; Wells et al. 2020) has suggested that ZCWPW1, which is recruited to PRDM9-bound sites, likely by its recognition of the H3K4me3 and H3K36me3 histone modifications deposited upon PRDM9 binding, may play an important role in this process.

Our previous work used genetic manipulation to modify the binding pattern of *M. musculus domesticus* PRDM9 (C57BL/6) to that of a common human PRDM9 sequence (hereafter, the humanized allele). When the modified allele was introduced into a sterile hybrid *M. musculus domesticus*×*M. musculus musculus* background, DSB sites moved to the binding sites of the humanized allele, and restoration of synapsis and fertility resulted (Davies et al. 2016). Molecular analysis that allows meiotic DSB sites to be mapped confirmed that the improved synapsis correlated with increased levels of PRDM9 binding to both homologs at matching positions. Like the humanized allele, new alleles arising in populations will likely recognize novel noneroded DNA sequences, present on both chromosome homologs, thus restoring chromosome pairing and fertility (fig. 1*c*).

Recent work has examined the effect of the humanized *Prdm9* allele in various intersubspecific hybrids, and its introduction was found to consistently improve the rate of chromosomal synapsis and restore fertility (Mukaj et al. 2020). To verify whether our previous finding that this restoration of fertility is likely due to increased binding of PRDM9 at matching positions on both homologous chromosomes in one of these additional humanized hybrids, we mapped the position of DSB sites in the hybrid male generated by a mating between STUS/Jpia (Pialek et al. 2008) (*M. musculus musculus*, hereafter STUS) and C57BL/6J (*M. musculus domesticus*, hereafter B6) mice, heterozygous for the humanized *Prdm9* allele. This was achieved using DMC1 ssDNA sequencing (SSDS), a modified chromatin immunoprecipitation (ChIP)-seq protocol which allows the position of a resected DSB to be mapped (Khil et al. 2012) (supplementary table 1, Supplementary Material online). We were able to establish which chromosome was being cut (Davies et al. 2016) and which *Prdm9* allele was responsible (Hinch et al. 2019) (supplementary fig. 1, Supplementary Material online). This allowed a metric to be calculated, measuring the extent to which evolutionary erosion has impacted each hotspot, resulting in PRDM9 binding and DSBs mainly on only one homolog (as defined by >75% of sequencing reads resulting from one homolog).

In the infertile STUSB6F1 hybrid, a large proportion (∼80% for both alleles) of the recombination sites for each allele occurred from such eroded hotspots, with only ∼20% resulting from positions where both homologs were being bound and cut. Replacement of the B6 allele by the humanized allele led to a substantial increase (to 66.5%) in the proportion of humanized PRDM9-controlled recombination sites occurring at such positions (supplementary fig. 2*a*, Supplementary Material online). This increased binding of the de novo humanized allele at matched positions on the two homologs correlated with substantially increased levels of synapsis (*P *<* *0.0001; *t*-test) and restored sperm production (*P* < 0.0001; *t*-test) to levels comparable with the parental strains (*P *>* *0.05; *t*-test) (supplementary fig. 2*b*–*f*, Supplementary Material online). This establishes reversal of evolutionary erosion as a likely cause of fertility rescue in these STUSB6F1 hybrids, consistent with our previous results in PWDB6F1 hybrids harboring the humanized *Prdm9* allele (Davies et al. 2016).

Having confirmed that the introduction of a de novo *Prdm9* allele rescues intersubspecific hybrid infertility, we next considered the impact of the de novo allele in a sterile hybrid generated from different species. The Algerian mouse, *M. spretus* (SPRET/EiJ; Dejager et al. 2009, hereafter SPRT), is a distinct species of mouse inhabiting south-western Europe, Morocco, and the western Mediterranean coast of Africa and male hybrids with *M. musculus* strains are known to be sterile (Guénet and Bonhomme 1981). The hybrid sterility has previously been characterized in some detail, and these studies have revealed a number of different mechanisms at play. Cytogenetic analyses found significant anomalies in synapsis including frequent X–Y chromosome asynapsis (Matsuda et al. 1991; Hale et al. 1993), with the majority of cells undergoing arrest during the first meiotic division. The fertility of hybrid females allowed backcrossing and subsequent genetic mapping of the infertility traits, identifying an involvement of chromosome 17 (Hammer et al. 1989; Pilder et al. 1991) and regions of the X-chromosome (Elliott et al. 2001). The X–Y asynapsis trait was strongly associated with loci within the pseudoautosomal region (Guénet et al. 1990; Matsuda et al. 1991; Hale et al. 1993). Chromosome 17 harbors the *Hst1* locus, now known to be *Prdm9* (Mihola et al. 2009), and X-chromosomal loci have long been associated with aspects of hybrid sterility (Storchová et al. 2004; Lustyk et al. 2019).

To assess the impact of de novo *Prdm9* alleles on these infertility phenomena, male SPRT mice were crossed with female B6 mice, heterozygous for either the humanized *Prdm9* allele or for an additional engineered allele in which the DNA-binding array of *Prdm9* is replaced with the orthologous sequence from the *M. musculus castaneus* subspecies (hereafter CAST) (Baker et al. 2014). Genetic modification of the DNA-binding array had no impact on *Prdm9* expression levels (supplementary fig. 3, Supplementary Material online).

Recombination hotspots were mapped by DMC1 SSDS and the cut chromosome and the responsible *Prdm9* allele determined in each of the hybrids (supplementary table 1 and supplementary fig. 4, Supplementary Material online). Only 15.0% of DMC1 sequence reads resulting from B6 PRDM9-controlled recombination sites in the wild-type B6SPRTF1 occurred at positions where both homologs were being appreciably bound and cut. In contrast, replacing the B6 allele with the humanized or CAST alleles improved this proportion over threefold, with 46.7% and 59.6% being observed for the de novo allele in the hybrids inheriting the humanized and the CAST alleles, respectively (fig. 2*a*). The hotspots occurring in the sterile B6SPRTF1 revealed a clear dominance of SPRT PRDM9 over B6. With the de novo alleles present, the dominance of the SPRT allele was reduced, with a stronger effect seen for the CAST allele (fig. 2*b*). A similar pattern of dominance was seen when assaying binding using the H3K4Me3 mark deposited by PRDM9 (fig. 2*b*).

**Fig. 2:**
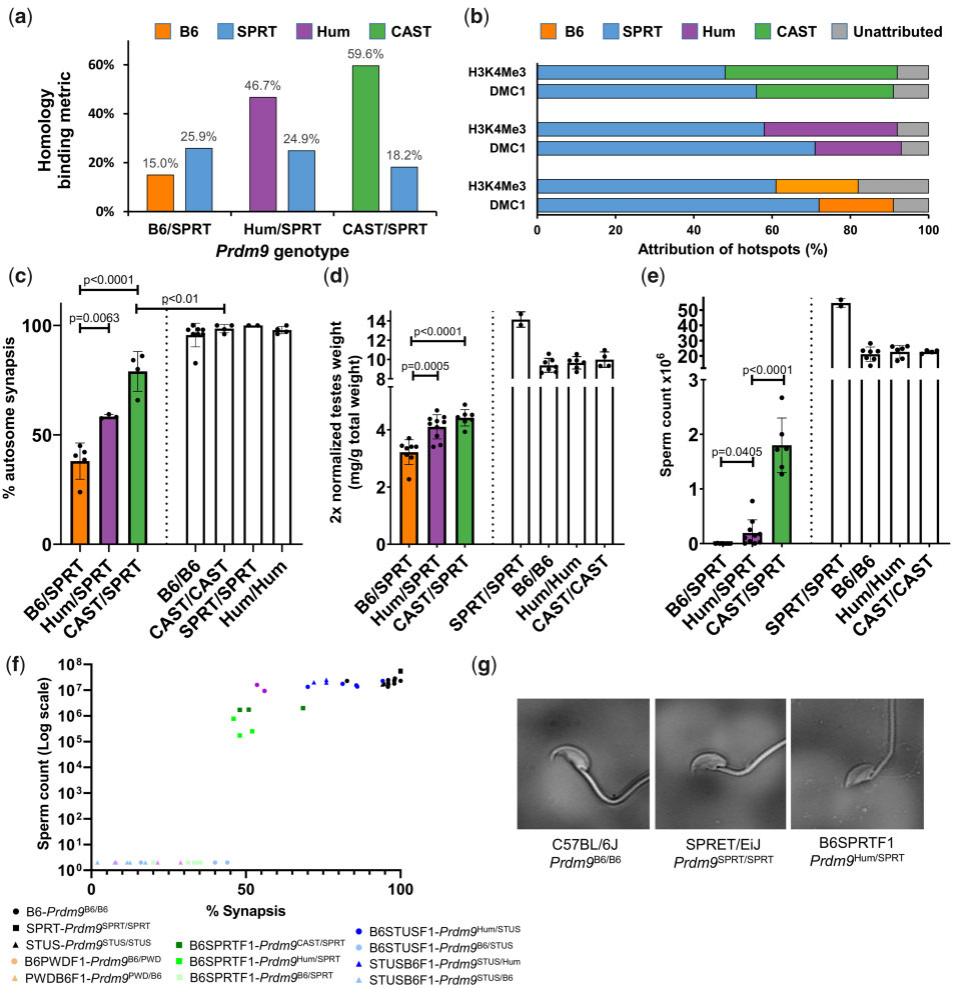
The introduction of de novo *Prdm9* alleles partially rescues fertility across the species barrier. (*a*) The proportion of DMC1 SSDS reads originating from matched positions on the two homologs (as defined by B6 contribution between 25% and 75%) for each of the alleles (SPRT: blue, B6: orange, Humanized: purple, CAST: green) present in B6SPRTF1 hybrids harboring the wild-type *Prdm9*^B6^ allele (B6/SPRT) or the engineered *Prdm9*^HUM^ (Hum/SPRT) and *Prdm9*^CAST^ (CAST/SPRT) alleles. (*b*) Overall hotspot attribution in these respective hybrids (bottom to top) using DMC1 ssDNA and H3K4Me3 ChIP sequencing peaks, colored as (*a*) and using gray for unattributed hotspots. (*c*) Mean proportion of normal autosomal synapsis (*n*=4), (*d*) testis weight (B6/SPRT, *n*=8; Hum/SPRT, *n*=10; CAST/SPRT, *n*=7), and (*e*) total sperm count (B6/SPRT, *n*=8; Hum/SPRT, *n*=10; CAST/SPRT, *n*=6) in the three hybrids and parental controls. Error bars show 1 SD. (*f*) The synapsis rate as determined by SYCP3/HORMAD2 staining, versus epididymal sperm count, are plotted for individual mice. PWDB6F1 and B6PWDF1 data are from Davies et al. (2016). An arbitrary sperm count of 2 has been given to mice with no sperm to enable the plot. (*e*) Morphology of representative sperm is shown for wild-type C57BL/6J mice, wild-type SPRET/EiJ mice, and the humanized hybrid, B6SPRTF1.

Cytological analysis of meiotic chromosomes revealed that levels of chromosomal autosomal synapsis were significantly improved by the introduction of either the humanized (*P *<* *0.01; *t*-test) or CAST (*P *<* *0.001; *t*-test) *Prdm9* allele, consistent with the improved binding of these de novo alleles on both homologs (fig. 2*c* and supplementary fig. 5, Supplementary Material online). Introduction of the CAST allele resulted in a higher synapsis rate than the humanized allele, consistent with the relative levels of improved binding across the homologs. The rescue of the asynapsis phenotype, however, was not complete.

In agreement with previous work (Matsuda et al. 1991, 1992; Hale et al. 1993), a high level of failure to synapse the X and Y chromosomes was evident in B6SPRTF1 hybrids relative to wild-type parental strains (*P *<* *0.01; *t*-test), and in contrast to the autosomes, the introduction of either of the de novo *Prdm9* alleles had no impact on X–Y asynapsis levels (*P *>* *0.05; *t*-test; supplementary fig. 6, Supplementary Material online). As expected, wild-type B6SPRTF1 hybrids were completely sterile, yet B6SPRTF1 hybrids harboring either the humanized or the CAST *Prdm9* allele showed significantly increased testes weight (*P *<* *0.001; *t*-test) (fig. 2*d*), and mature spermatozoa were recovered from the epididymis, albeit in much lower numbers than in the parental strains (fig. 2*e*). Introduction of the CAST allele resulted in a more pronounced rescue than the humanized allele, as reflected by significantly higher testes weight and sperm count (*P *<* *0.0001; *t*-test), which also correlated with the larger impact on homologous binding seen for the CAST allele.

Our investigations of wild-type and genetically engineered hybrid mice reveal a spectrum of autosomal synapsis rates and fertility. By examining these parameters in individual mice, an association between synapsis rate and spermatozoa production was revealed which suggests a “cliff-edge” rather than a smoothly increasingly relationship, with a certain threshold of synapsis—approximately 50%—being required for any significant level of sperm production to occur (fig. 2*f*). Below this threshold, there is little or no sperm production. Above the threshold, sperm production is substantial. This observation is in accord with previous studies: a requirement of at least 50% synapsis of the sex chromosomes to achieve sperm production was revealed by a study examining mice expressing the *Spo11β* isoform with varying genetic background (Faisal and Kauppi 2016). Similarly, an approximately 50% threshold was also evident in studies of *M. musculus*×*M. domesticus* hybrids (Mukaj et al. 2020). A possible explanation for this relationship lies in the ability of spermatocytes to exchange cytoplasmic products via cytoplasmic bridges, and indeed apoptopic spermatocytes have been shown to eliminate nearby unaffected cells, presumably because of these bridges (Royo et al. 2010; ElInati et al. 2017). Potentially this bystander effect, or nonindependent cell death, explains the sudden dramatic effect on spermatozoa production when asynapsis reaches some critical level.

Although appreciable levels of sperm production were restored in this interspecies hybrid, the rescued hybrids were still unable to sire litters in natural matings. Examination of the rescued sperm revealed an aberrant head morphology, with the characteristic spike, present in both parental strains, being absent (fig. 2*g*). In vitro fertilization (IVF) was performed successfully with sperm recovered from the humanized B6SPRTF1 males only when the zona pellucida of the oocytes was removed (supplementary fig. 7, Supplementary Material online). We infer that the aberrant sperm head was incompatible with the sperm’s ability to adhere or traverse the zona pellucida. Fertilized embryos were transferred into a recipient female and a litter of two pups resulted, genetic analysis of which confirmed that one pup had been derived from IVF with the humanized B6SPRTF1 sperm, confirming the viability of the rescued sperm (supplementary fig. 7, Supplementary Material online).

Despite the persistence of these additional reproductive blocks, the data establish for the first time that PRDM9 is, and remains, a key but reversible part of the hybrid infertility between these two species. Introduction of de novo *Prdm9* alleles increases instances where PRDM9 binds at the same position on both chromosome homologs. This, in term, increases the efficiency of homology search, increasing the rate of synapsis above a critical threshold, enabling the completion of meiosis and the partial restoration of sperm production. This partial restoration allows the two further reproductive blocks to be more clearly revealed.

Firstly, the introduction of de novo *Prdm9* alleles ameliorates the autosomal asynapsis seen in the B6SPRTF1 hybrids, yet the asynapsis of the sex chromosomes remains evident and does not appear to be influenced by *Prdm9*. Indeed, early genetic mapping studies have implicated the distal region of the X-chromosome in this phenomenon, and not *Hst1* (*Prdm9*), suggesting an incompatibility between the pseudoautosomal regions (PAR) of the two species is responsible (Guénet et al. 1990; Matsuda et al. 1991; Hale et al. 1993). Structural constraints may underlie these genetic signals, as differences in the PAR boundary and the composition of the PAR sequences have been reported between *M. spretus and M. musculus* subspecies (White, Ikeda, et al. 2012; Morgan et al. 2019)*.* PAR asynapsis being *Prdm9*-independent is consistent with previous findings that PRDM9-dependent recombination plays a relatively small role in PAR recombination in mice (Brick et al. 2012; Hinch et al. 2014).

Secondly, in the hybrids harboring de novo *Prdm9* alleles, sperm production occurs in the B6SPRTF1 hybrid, but the sperm show aberrant head morphology. Histological examination of B6SPRTF1 testes sections had previously revealed rare cases of spermatocytes which progress beyond the first metaphase developing abnormally with head shape anomalies (Matsuda et al. 1991). The increased production of spermatozoa in hybrids harboring de novo *Prdm9* alleles, with appreciable numbers obtainable from the caudal epididymis, makes this phenotype abundantly clear. Aberrant sperm head morphology has been reported for interspecific crosses between *M. musculus and M. macedonicus* (Elliott et al. 2004) and is a frequently observed phenomenon in intersubspecific hybrids, with genetic mapping studies implicating several loci (Storchová et al. 2004; White et al. 2011; Campbell et al. 2012; White, Stubbings, et al. 2012). Sperm head abnormalities have previously been shown to correlate with aberrant gene expression programs during spermatogenesis resulting from perturbations in the processes which maintain transcriptional silencing of the sex chromosomes, particularly through postmeiotic sperm development (Cocquet et al. 2009; Larson et al. 2017). Matched expression of the Y-chromosomal multicopy gene *Sly*, and its related X-chromosomal paralog, *Slx*, are essential for this process (Ellis et al. 2005). Interestingly, *Slx and Sly* copy numbers are usually well correlated within a species, but significant copy number variation exist between *M. spretus and M. musculus* (Morgan and Pardo-Manuel de Villena 2017). Furthermore, experimentally induced reduction in *Sly* has been shown to result in spermatozoa of abnormal morphology (Cocquet et al. 2009), highlighting the need for balanced expression of these related genes. Whether the sperm abnormalities result from a breakdown in postmeiotic sex chromatin repression due to mismatched *Slx and Sly* expression in B6SPRTF1 or whether failure in these silencing mechanisms also arises due to the observed meiotic X–Y asynapsis will demand further investigation. Abnormal head morphology has previously been shown to be incompatible with oocyte penetration and fertilization (Krzanowska and Lorenc 1983), and our results confirm that fertilization with the abnormal sperm can only be achieved by IVF in oocytes stripped of their zona pellucida.

Besides PRDM9’s role in regulating the efficiency of autosomal synapsis, this study and others (Oka et al. 2010) highlight multiple additional factors that may contribute to the sterility of *M. spretus and M. musculus* interspecies male hybrids. Our genetic approach of relaxing the constraint imposed by PRDM9 will allow further genetic dissection of reproductive barriers at play in these interspecific hybrids.

In summary, our results establish that the introduction of de novo alleles on the B6SPRTF1 background increases the binding of PRDM9 at the same position on both homologs, allowing more efficient homology search and rapid repair of DSBs. This increases the chromosome synapsis rate toward a critical threshold, allowing the completion of meiosis and the generation of mature sperm capable of generating offspring. With manipulation of a single gene, we are able to rescue aspects of the infertility that has arisen between two species separated by 1.5 My of evolution (Boursot et al. 1993).

## Materials and Methods

### Animals

Genetically modified mice harboring the human PRDM9 B allele (*Prdm9*^tm1.1(PRDM9)Wthg^) were generated in house (Davies et al. 2016), mice harboring the *M. musculus castaneus Prdm9* allele (Baker et al. 2014) (*Prdm9*^tm1.1Kpgn^) were provided by Petko Petkov (Jackson Laboratories, Bar Harbor). Wild-type STUS/Jpia mice were provided by Jaroslav Piálek, Institute of Vertebrate Biology, Brno and wild-type *M. spretus* mice (SPRET/EiJ) mice were obtained from MRC Harwell. *Gt(ROSA)26Sor*^tm1(CAG-cas9)Wthg^ were generated in house (Cebrian-Serrano et al. 2017). The breeding of the hybrid mice was carried out in accordance with UK Home Office Animal (Scientific Procedures) Act 1986, with procedures reviewed by the Clinical Medicine Animal Welfare and Ethical Review Body at the University of Oxford, and conducted under project license PPL 30/3437 and 30/3085. Animals were housed in individually ventilated cages, provided with food and water ad libitum and maintained on a 12 h light:12 h dark cycle (150–200 lx). The only reported positives on FELASA health screening over the entire time course of these studies were for *Helicobacter hepaticus and Entamoeba* spp. Experimental groups were determined by genotype and were therefore not randomized, with no animals excluded from the analysis. Sample sizes for fertility studies were selected on the basis of previously published studies (Davies et al. 2016) and all phenotypic characterization was performed blind to experimental group.

Heterozygous mice harboring the human allele were bred with STUS/Jpia in both directions (using females and males of each type) to generate the STUSB6F1 and B6STUSF1 hybrids. Heterozygous female mice harboring either the human or the CAST *Prdm9* alleles were bred with male SPRET/EiJ mice to generate the B6SPRTF1 hybrids. Male hybrid offspring were analyzed at 10 weeks of age. Paired testes weight from individual mice was recorded and normalized against lean body weight, as assessed using EchoMRI-100 Small Animal Body Composition Analyzer. Sperm count was obtained from individual mice by allowing caudal sperm to swim out in 1,000 μl of warm PBS prior to counting with an hemocytometer.

### Hotspot Determination and Provenance

Chromatin immunoprecipitation with a rabbit polyclonal anti-DMC1 antibody (Santa Cruz Biotechnology, sc-22768) followed by SSDS was performed as previously described (Khil et al. 2012) with some modifications (Hinch et al. 2019). ChIP-seq with a rabbit polyclonal anti-H3K4me3 antibody (Abcam ab8580) was performed as previously described (Li et al. 2019). Hotspot centers were localized by running a bioinformatic pipeline for identification of single-stranded sequences (Khil et al. 2012) followed by de novo calling using a previously published peak calling algorithm (Davies et al. 2016). Over 10,000 hotspots were called de novo in each of the DMC1 assays reported (supplementary table 1, Supplementary Material online) and the analyses presented are based on the full set of autosomal hotspots. The PRDM9 allele responsible for activating specific hotspots was identified by comparison with mice harboring a range of distinct alleles, as previously described (Hinch et al. 2019). PRDM9 DNA motifs were inferred using a previously published Bayesian, ab initio motif finding algorithm (Altemose et al. 2017). For each hotspot in the hybrid mouse, the fraction of reads that originated from the B6 and the SPRET (or STUS) chromosomes respectively, was inferred as previously described (Davies et al. 2016). For mapping of reads to homologous chromosomes in B6SPRTF1, we leveraged previously published variant calls for SPRET/EiJ by the Mouse Genomes Project (MGP version 5 Release 1505) (Keane et al. 2011). We restricted to SNPs that passed all MGP filters and we further filtered out SNPs that were heterozygous in SPRET/EiJ. To perform this analysis in B6xSTUS hybrids, we first sequenced a male STUS mouse to an average depth of 21× and used Platypus (Rimmer et al. 2014) version 0.5.2 to call variants. We restricted to SNPs that passed all quality filters and were homozygous in this animal.

### Immunocytochemistry

Mouse testis chromosome spreads were prepared using surface spreading and immunostained as previously described (Davies et al. 2016). The following primary antibodies were used: mouse anti-SYCP3 (Santa Cruz Biotechnology sc-74569, D-1) and rabbit anti-HORMAD2 (Santa Cruz Biotechnology sc-82192), and Alexa Fluor 594- or 488-conjugated secondary antibodies against rabbit or mouse IgG, respectively (ThermoFisher Scientific). Images were acquired using either a BX-51 upright wide-field microscope equipped with a JAI CVM4 B&W fluorescence CCD camera and operated by the Leica Cytovision Genus software, or a Leica DM6B microscope for epifluorescence, equipped with a DFC 9000Gt B&W fluorescence CCD camera, and operated via the Leica LASX software. Image analysis was carried out using Fiji (ImageJ-win64). Synapsis rate was estimated by the analysis of at least 50 meiotic nuclei. For synapsis analysis of the sex chromosomes, following immunostaining of spermatocytes, fluorescent in situ hybridization identification of the PAR was achieved with probes labeled with the Nick translation Kit (Abbott Molecular) as follows:

Chr X: RP23-119M14 + RP23-168A19 directly labeled with Gold dUTP (Enzo LifeScience); PAR region: RP24-500I4, directly labeled with Aqua dUTP (Enzo LifeScience); Chr Y: bMQ53i13, labeled with digoxigenin dUTP (Sigma Aldrich), detected with sheep antidigoxigenin (Sigma Aldrich), and antisheep 633 (ThermoFisher Scientific).

### Assisted Reproduction

In vitro fertilization was performed as previous described (Takeo and Nakagata 2011). Briefly, sperm was dispersed in a modified Krebs-Ringer bicarbonate solution (TYH) containing 1.0 mg/ml of polyvinyl alcohol and 0.75 mM methyl-β-cyclodextrin (Sigma), before transferring to fertilization drops containing cumulus masses of C57BL/6J oocytes in human tubal fluid medium (Millipore) supplemented with 0.25 mM reduced l-glutathione (Sigma)and 4 mg/ml Bovine Serum Albumin. After 4 h of incubation, presumptive zygotes were washed through drops of human tubal fluid and incubated in vitro in KSOM media (Millipore), supplemented with 0.8 mg/ml Bovine Serum Albumin (Takeo and Nakagata 2011). Where required, zona pellucida was removed by incubating oocytes briefly in acidic Tyrode’s solution (Sigma Aldrich). The resulting zygotes were cultured to the morula/blastocyst stage or were transferred at the two-cell stage surgically into pseudopregnant CD1 recipients. Where required, IVF-derived embryos were supplemented with additional heterozygous *B6.129S6-Gt(ROSA)26Sor^tm1(CAG-cas9)Wthg^* embryos to allow a suitable number for embryo transfer. Microsatellite analysis was performed by PCR using primer sets *D12Mit136 and D12MIt14.*

## Supplementary Material

Supplementary data are available at *Molecular Biology and Evolution* online.

## Supplementary Material

msab269_Supplementary_Data
