# Supplementary material for: Altering the Binding Properties of PRDM9 Partially Restores Fertility across the Species Boundary
Source: Mol Biol Evol. 2021 Sep 7;38(12):5555–62. doi: 10.1093/molbev/msab269 (PMC8662609; doi:10.1093/molbev/msab269)
Supplement: msab269_Supplementary_Data [file msab269_supplementary_data.zip › Supplementary Figures 2nd August.pdf]

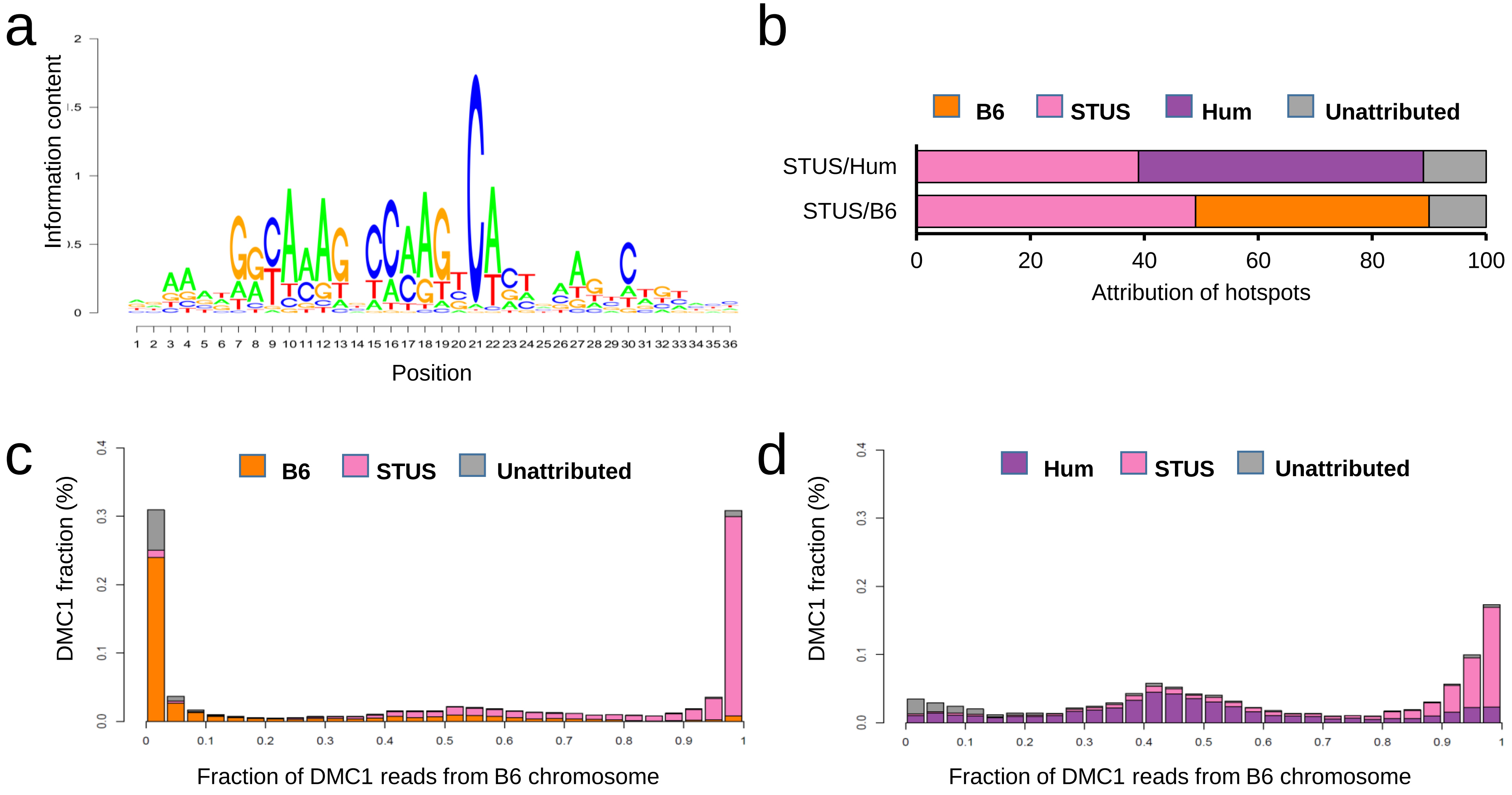

Supplementary Fig. 1 – DMC1 ssDNA sequencing of humanized and wild-type STUSB6F1 mice.
a) STUS hotspot consensus PRDM9 binding motif as inferred from hotspot overlap between B6STUSF1-*Prdm9*$^{B6/STUS}$ and B6STUSF1-*Prdm9*$^{Hum/STUS}$. **b)** Overall hotspot attribution in the STUSB6F1 harbouring the wild-type *Prdm9*^{B6} allele (STUS/B6) or the engineered *Prdm9*^{HUM} allele (STUS/HUM) using DMC1 ssDNA sequencing (SSDS) peaks. **c)** Distribution of the fraction of DMC1 SSDS reads originating from the B6 chromosome in the infertile STUS/B6 mouse and **d)** for the fertile STUS/Hum mouse. PRDM9-control at each hotspot is attributed to B6 (orange), Humanized (purple), STUS (pink) or undetermined (grey) alleles.

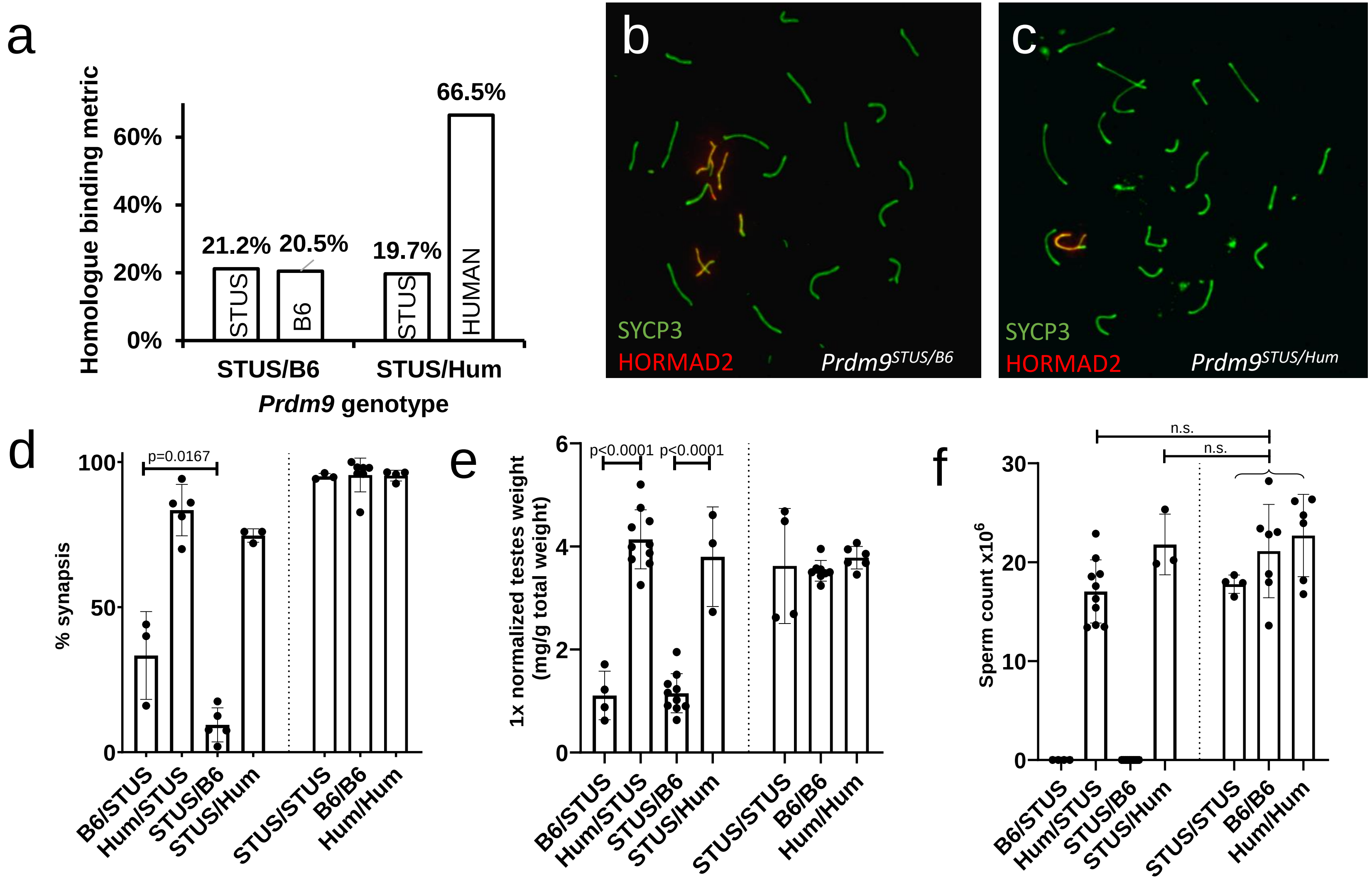

Supplementary Fig. 2 - Introduction of a humanized *Prdm9* allele rescues the sterility phenotypes associated with STUSB6F1 and B6STUSF1 hybrids.
a) The proportion of DMC1 SSDS reads originating from matched positions on the two homologues (as defined by B6 contribution between 25-75%) for each of the alleles present in STUSB6F1 hybrids harbouring the wild-type *Prdm9*^{B6} allele (STUS/B6) or the engineered *Prdm9*^{HUM} allele (STUS/Hum). **b)** and **c)** Representative immunofluorescence staining of testis nuclear spreads from wild-type (b) and humanized STUSB6F1 (c) for the synaptonemal complex protein SYCP3 which labels the chromosome axis (green) and HORMAD2 which marks unsynapsed chromosomes (red). A pronounced asynapsis of autosomes is typical of wild-type STUSB6F1, whereas the STUSB6F1 hybrids harbouring the humanized *Prdm9* allele typically reveal complete synapsis. **d)** Proportion of cells with normal autosomal synapsis, **e)** testis weight and **f)** total sperm count for the wild-type (B6/STUS; n=4) and humanized (Hum/STUS; n=10) B6STUSF1 hybrids, the wild-type (STUS/B6; n=10) and humanized (STUS/Hum; n=3) STUSB6F1 hybrids and the parental strains. Error bars show 1 s.d.

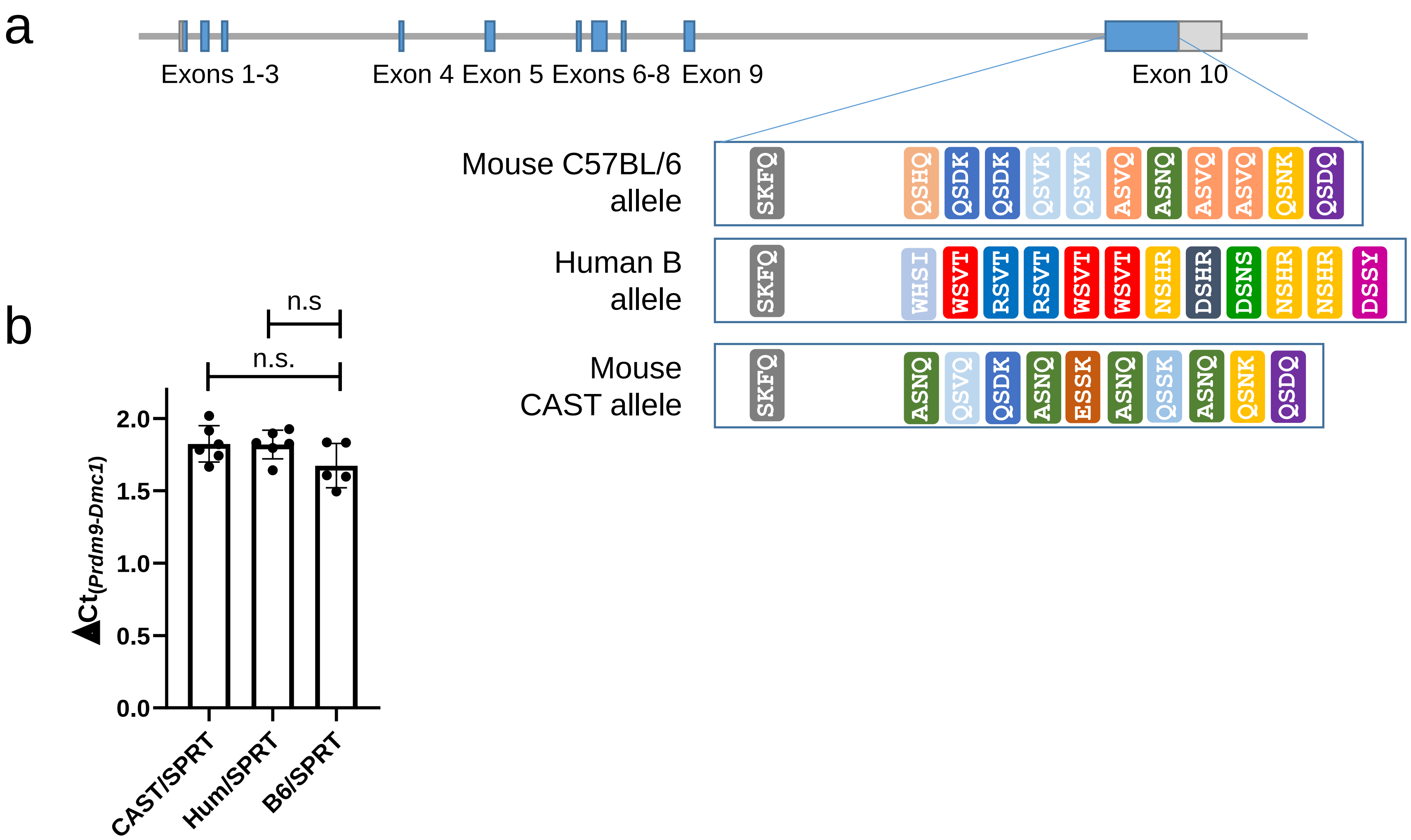

Supplementary Fig.3: Expression of *Prdm9* is unchanged by the introduction of *de novo Prdm9* alleles in the B6SPRTF1 hybrid

a) Structure of the B6, humanized and CAST alleles used in this study. The enlargement of the DNA-binding zinc finger array is shown with each zinc finger labelled by the 4 predicted DNA-contacting amino acids **b)** Expression of the *Prdm9* transcript, normalised to *Dmc1* expression, from testis cDNA prepared from wild-type B6SPRTF1-*Prdm9*$^{B6/SPRT}$ (B6/SPRT; n=5), B6SPRTF1-*Prdm9*$^{Hum/SPRT}$ (Hum/SPRT; n=6) or B6SPRTF1-*Prdm9*$^{CAST/SPRT}$ (CAST/SPRT; n=6). No significant difference in expression could be found for any of the *de novo Prdm9* alleles. Error bars show 1 s.d.

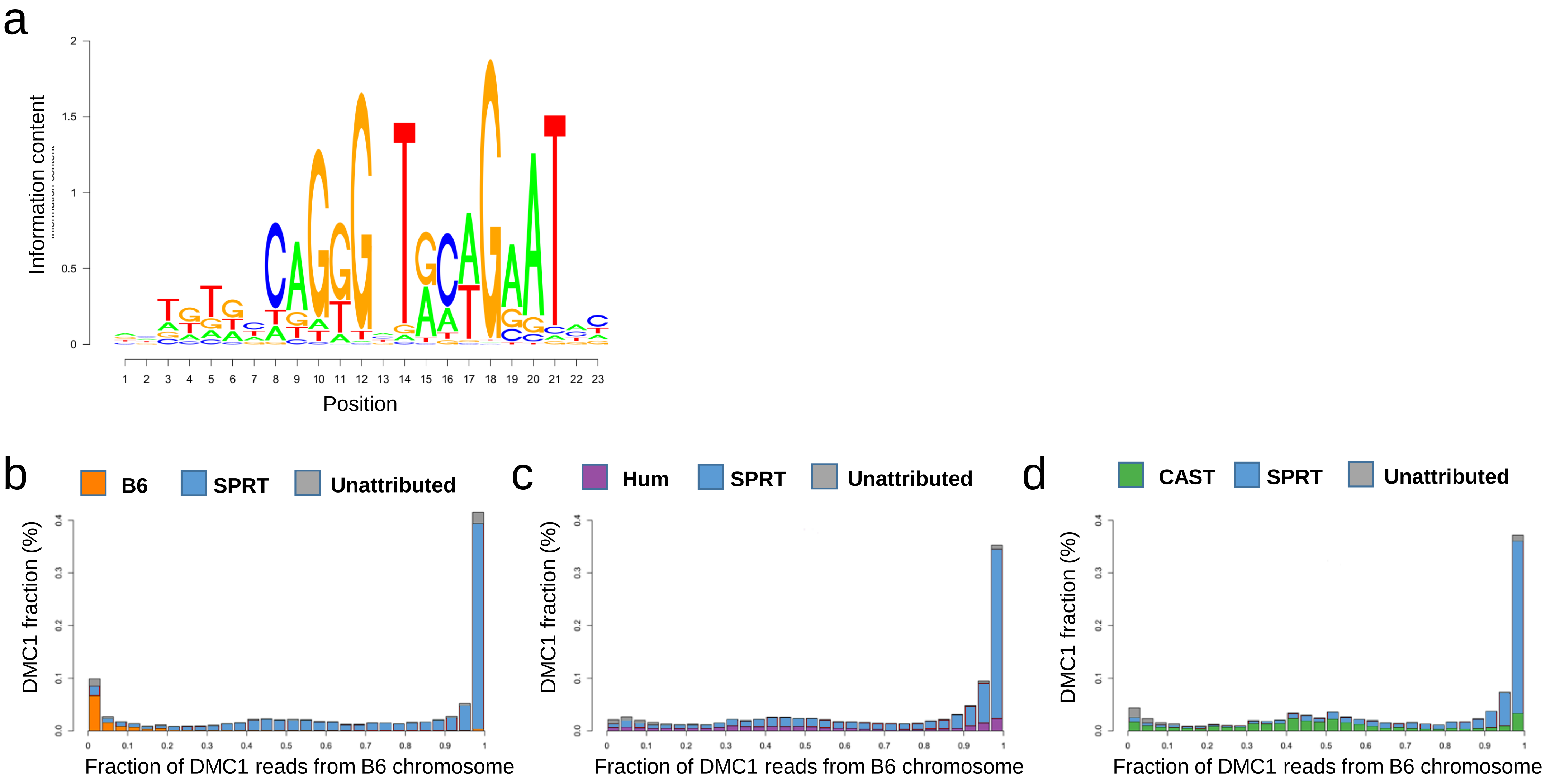

Supplementary Fig.4 – DMC1 ssDNA sequencing of B6SPRTF1 hybrid mice harbouring wild-type and engineered *de novo Prdm9* alleles.
a) SPRT hotspot consensus PRDM9 binding motif as inferred from hotspot overlap between B6SPRTF1-*Prdm9*$^{B6/SPRT}$ and B6SPRTF1-*Prdm9*$^{Hum/SPRT}$. **b), c), d)** Distribution of the fraction of DMC1 SSDS reads originating from the B6 chromosome in the B6SPRTF1 mouse harbouring **b)** *Prdm9*^{B6}, **c)** *Prdm9*^{Hum} and **d)** *Prdm9*^{CAST}. PRDM9-control at each hotspot is attributed to B6 (orange), Humanized (purple), SPRT (blue), CAST (green) or undetermined (grey) *Prdm9* alleles.

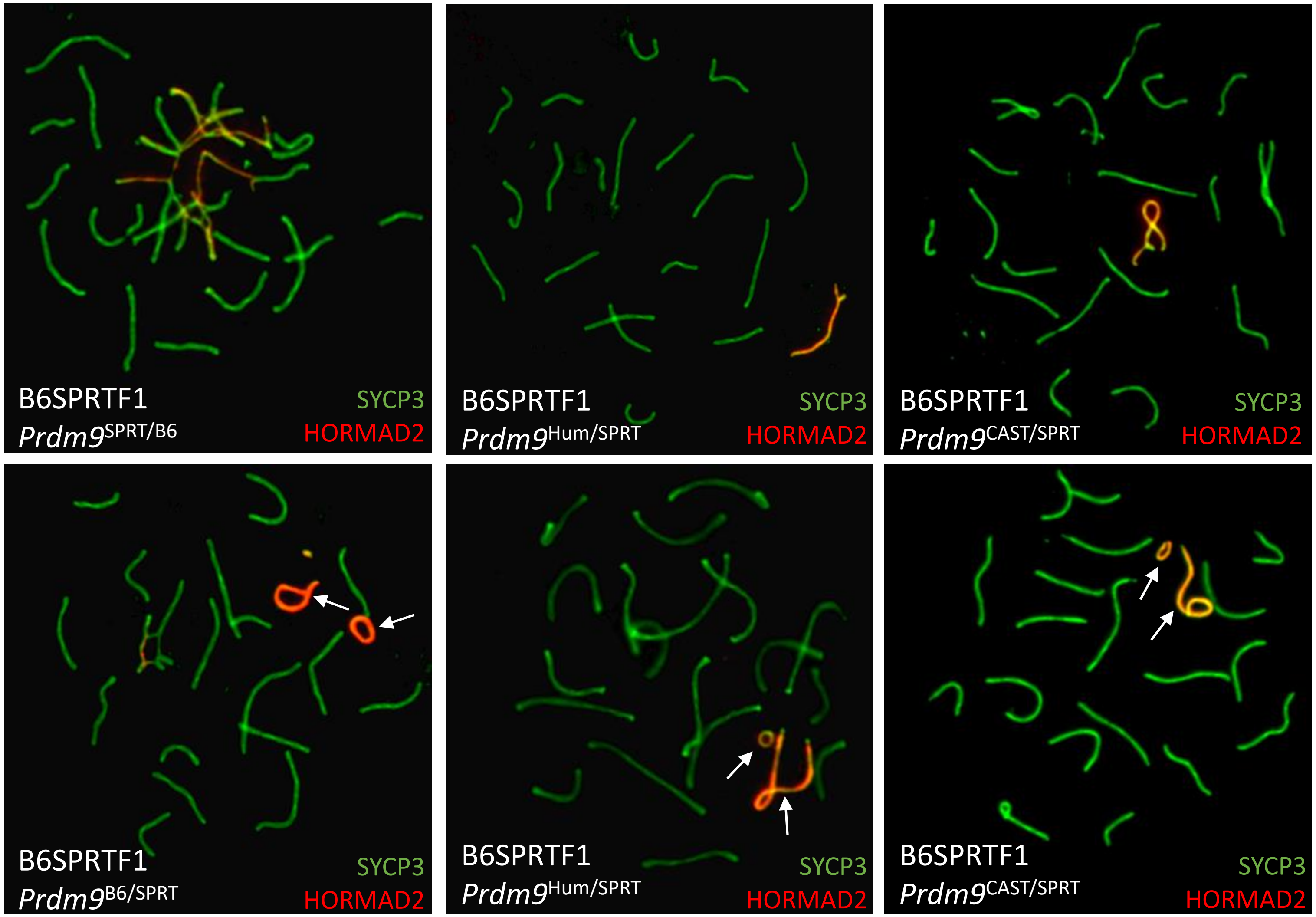

Supplementary Fig.5: Representative micrographs demonstrating the synapsis status in the B6SPRTF1 hybrids

Representative immunofluorescence staining of testis nuclear spreads from wild-type B6SPRTF1-*Prdm9*$^{B6/SPRT}$ (left panels), B6SPRTF1-*Prdm9*$^{Hum/SPRT}$ (middle panels), or B6SPRTF1-*Prdm9*$^{CAST/SPRT}$ (right panels) for the synaptonemal complex protein SYCP3 which labels the chromosome axis (green) and HORMAD2 which marks unsynapsed chromosomes (red). A pronounced asynapsis of autosomes is typical of wild-type B6SPRTF1, whereas the B6SPRTF1 hybrids harbouring the *de novo* alleles reveal higher levels of complete autosomal synapsis. In all genotypes, however, irrespective of autosomal synapsis status, asynapsis of the sex chromosomes is seen in approximately one fifth of cells (see Supplementary Fig. 6a) (lower panels, marked by arrows).

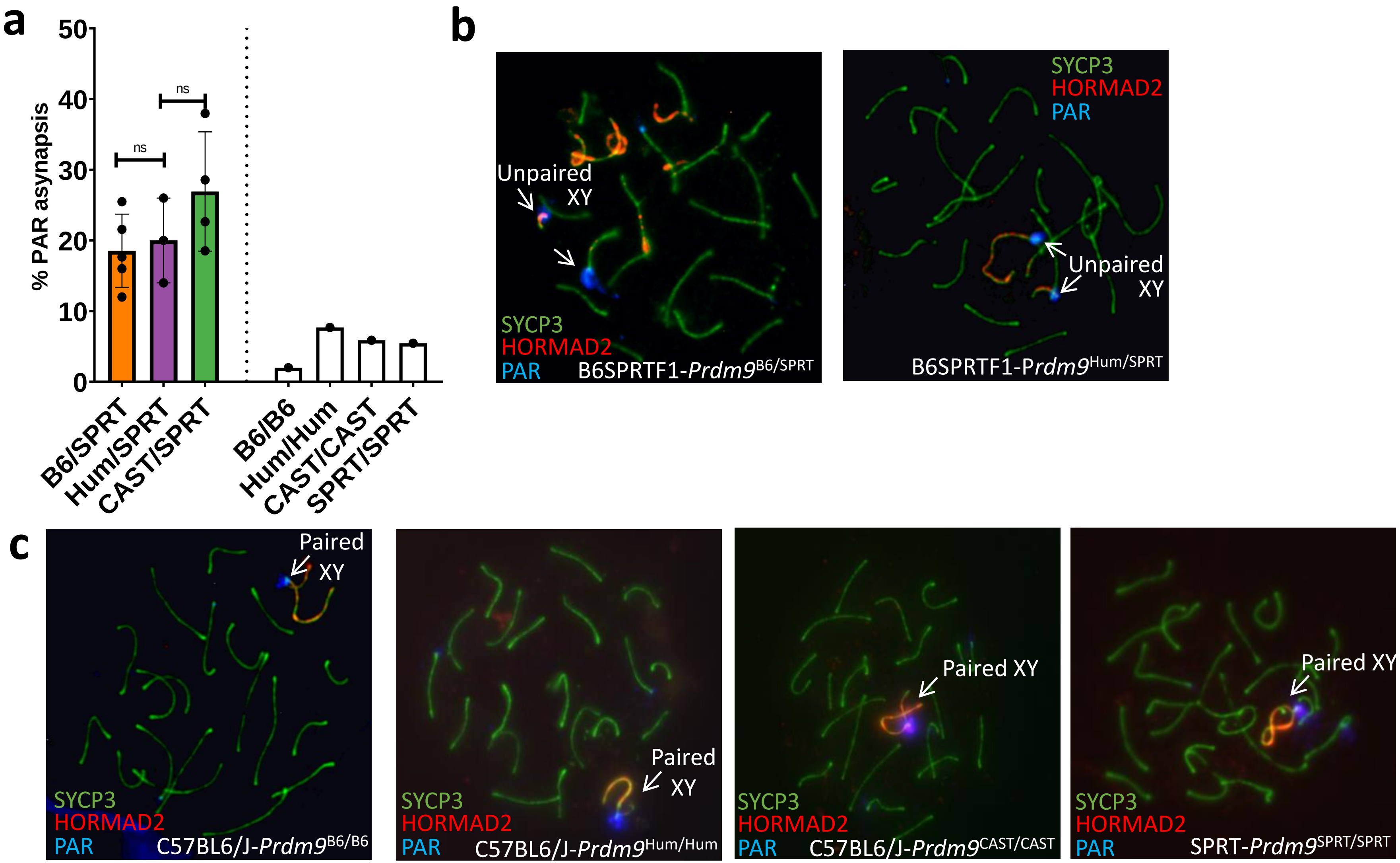

Supplementary Fig.6: Representative micrographs demonstrating PRDM9-independent sex-chromosome asynapsis in the B6SPRTF1 hybrids

a) Proportion of sex chromosome asynapsis in the wild-type B6SPRTF1-*Prdm9*$^{B6/SPRT}$ (B6/SPRT; n=5), B6SPRTF1-*Prdm9*$^{Hum/SPRT}$ (Hum/SPRT; n=3), B6SPRTF1-*Prdm9*$^{CAST/SPRT}$ (CAST/SPRT; n=4) and parental strains for reference. Error bars show 1 s.d. **b)** Representative immunofluorescence staining of testis nuclear spreads from B6SPRTF1-*Prdm9*$^{B6/SPRT}$ (left panel), or B6SPRTF1-*Prdm9*$^{Hum/SPRT}$ (right panel) for the synaptonemal complex protein SYCP3 which labels the chromosome axis (green) and HORMAD2 which marks unsynapsed chromosomes (red), plus the addition of a fluorescent *in situ* hybridization probe against the pseudoautosomal region (PAR, blue). **c)** as b) for the parental strains. The X-Y chromosomes and their paired status is shown by white arrows.

a

Experiment	Sperm	Treatment	No. of oocytes	2-cell	% fertilization
1	B6SPRTF1-Prdm9$^{Hum/SPRT}$		93	6	6%
	C57BL/6J		25	21	84%
2	B6SPRTF1-Prdm9$^{Hum/SPRT}$		56	0	0%
	C57BL/6J		31	30	97%
3	B6SPRTF1-Prdm9$^{Hum/SPRT}$		160	1	1%
	C57BL/6J		18	11	61%
4	B6SPRTF1-Prdm9$^{Hum/SPRT}$	Zona stripping	22	11	50%
	C57BL/6J	Zona stripping	10	7	70%
5	B6SPRTF1-Prdm9$^{Hum/SPRT}$	Zona stripping	80	9	11%
	C57BL/6J	Zona stripping	11	7	64%

b

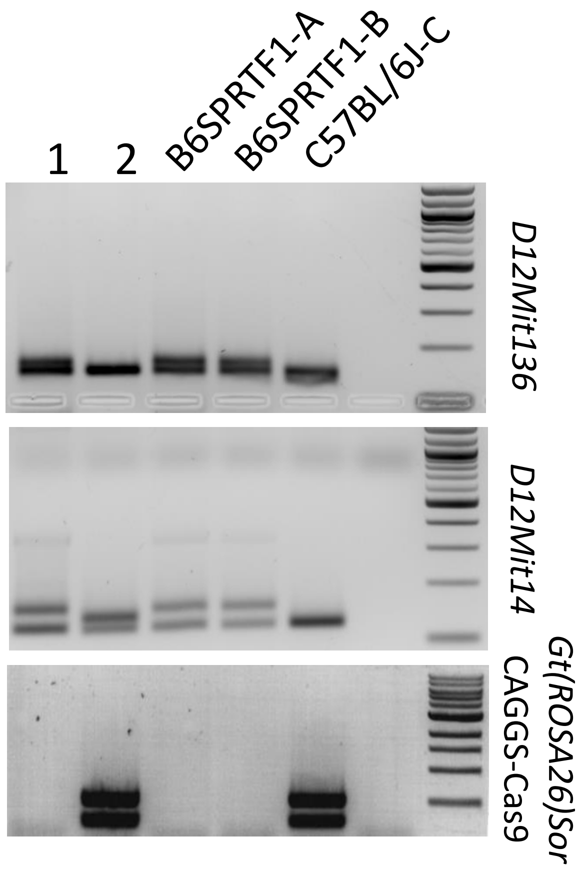

Supplementary Fig. 7: IVF using humanized B6SPRTF1 sperm

a) Table showing the fertilization rate of C57BL/6J oocytes with humanized B6SPRTF1-*Prdm9*$^{Hum/SPRT}$ sperm or C57BL/6J control sperm over a number of different experiments, with and without the stripping of the zona pellucida. Fertilization rate is determined by the number of 2-cell embryos obtained following overnight incubation. **b)** Top and middle panels show the genotyping of offspring #1 and #2 for two microsatellite markers (*D12Mit136* and *D12MIt14*) which distinguish between B6 and SPRT. Offspring #1 shows a characteristic doublet for the microsatellite genotyping, indicative of heterozygosity for B6 and SPRT at these markers (compare with the two control B6SPRTF1 mice [B6SPRTF1-A and B6SPRTF1-B] and a control C57BL/6J mouse). The lower panel shows the genotyping specific for the *Gt(ROSA)26Sor*$^{tm1(CAG\text{-}cas9)Wthg}$ allele, which genetically marks the additional embryos used to supplement the IVF embryos for embryo transfer to maximize the chance of pregnancy. Offspring #1 is negative for *Gt(ROSA)26Sor*$^{tm1(CAG\text{-}cas9)Wthg}$ and therefore must have been derived from the B6SPRTF1 IVF, whereas Offspring #2 is positive for this marker, and thus was derived from carrier embryos added to the embryo transfer. Additionally, offspring #2 carries a different allele at D12Mit136, reflecting 129S6 genetic background within the carrier embryos.

Sample	Total number of hotspots (called by DMC1 ChIP-seq)	Number of autosomal hotspots (subset of total number)
B6/SPRT	19247	18522
Hum/SPRT	16398	15727
CAST/SPRT	10088	9592
STUS/B6	18069	17309
STUS/Hum	10525	10031

Supplementary Table 1: DMC1 Hotspots called for each of the genotypes analysed
Table shows the number of DMC1 hotspots in total, and on autosomes, for the B6SPRTF1 mice with genotypes *Prdm9*$^{B6/SPRT}$, *Prdm9*$^{Hum/SPRT}$ and *Prdm9*$^{CAST/SPRT}$ and for the STUSB6F1 mice with genotypes *Prdm9*$^{STUS/B6}$ and Prdm9$^{\mathrm{STUS/Hum}}$.
